# The Relationship between *Helicobacter pylori* Infection of the Gallbladder and Chronic Cholecystitis and Cholelithiasis: A Systematic Review and Meta-Analysis

**DOI:** 10.1155/2021/8886085

**Published:** 2021-01-06

**Authors:** Liang Wang, Junyin Chen, Wenxi Jiang, Li Cen, Jiaqi Pan, Chaohui Yu, Youming Li, Weixing Chen, Chunxiao Chen, Zhe Shen

**Affiliations:** ^1^Department of Gastroenterology, The First Affiliated Hospital, College of Medicine, Zhejiang University, Hangzhou, China; ^2^Endoscopy Center, Cangzhou Central Hospital of Hebei Province, Cangzhou, Hebei Province, China; ^3^Department of Gastroenterology, The Affiliated Hospital, Shaoxing College of Arts and Sciences, Shaoxing, Zhejiang Province, China

## Abstract

*Helicobacter pylori* (*H. pylori*) is proved to be the main pathogenic agent of various diseases, including chronic gastritis, gastric ulcer, duodenal ulcer, and gastric cancer. In addition, chronic cholecystitis and cholelithiasis are common worldwide, which are supposed to increase the total mortality of patients. Epidemiologic evidence on the relationship between *H. pylori* infection of the gallbladder and chronic cholecystitis/cholelithiasis still remains unclear. We conducted a systematic review and meta-analysis of overall studies to investigate the relationship between *H. pylori* infection of the gallbladder and chronic cholecystitis/cholelithiasis. Two researchers searched PubMed, Embase, and Cochrane Library databases to obtain all related and eligible studies published before July 2020. The pooled odds ratios (ORs) and corresponding 95% confidence intervals (CIs) were calculated by the random-effects model. Subgroup analysis, heterogeneity, publication bias, and sensitivity analysis were also conducted. Twenty studies were included in the meta-analysis, involving 1735 participants and 1197 patients with chronic cholecystitis/cholelithiasis. *Helicobacter* species infection of the gallbladder was positively correlated with increased risk of chronic cholecystitis and cholelithiasis, especially *H. pylori* (OR = 3.05; 95% CI, 1.81–5.14; *I*^2^ = 23.5%). Besides, country-based subgroup analysis also showed a positive correlation between the gallbladder *H. pylori* positivity and chronic cholecystitis/cholelithiasis risk. For Asian and non-Asian country studies, the ORs were 4.30 (95% CI, 1.76–10.50; *I*^2^ = 37.4%) and 2.13 (95% CI, 1.23–3.70; *I*^2^ = 0.0%), respectively. The association was more obvious using the bile sample and urease gene primer. In conclusion, this meta-analysis provided evidence that there is a positive correlation between *H. pylori* infection in the gallbladder and increased risk of chronic cholecystitis and cholelithiasis.

## 1. Introduction

*Helicobacter pylori (H. pylori)* is a Gram-negative, spiral-shaped, and microaerophilic microorganism, which is proved to be the main pathogenic agent in the occurrence and development of chronic gastritis, gastric ulcer, and duodenal ulcer [1]. Furthermore, *H. pylori* is also associated with an increased risk of gastric malignant lymphoma of the mucosa-associated lymphoid tissue (MALT) as well as gastric adenocarcinoma [2–4].

Chronic cholecystitis and cholelithiasis are quite widespread worldwide. Geography and especially ethnicity play an important role in the prevalence of gallstone diseases. The prevalence rate in the west is higher than that in the east, ranging from 4% to 74% [5]. Recently, some studies indicated that people with gallstone diseases had increased total mortality and cardiovascular disease and cancer mortality [6, 7]. Besides, since chronic inflammation is caused by repeated trauma to the gallbladder mucosa and DNA damage led by high or abnormal bile acid exposure, gallstones are closely related to the development of hepatobiliary cancers, including gallbladder cancer [8]. Therefore, chronic cholecystitis and cholelithiasis constitute a serious health problem, which brings a great medical burden to the society.

Since Chang et al. [9] incidentally discovered *H. pylori* in the gallbladder's mucosa of a patient with cholecystitis in 1996, more and more studies have detected various *Helicobacter* species including *H. pylori*, *H. bilis*, *H. hepaticus*, *H. pullorum*, *and H. ganmani* in the gallbladder tissue, gallstone, and bile taken from the gallbladder [10, 11]. Among these various *Helicobacter* species, *H. pylori* is the commonest one. The high prevalence of *H. pylori* in the group of patients with chronic cholecystitis raises questions concerning about the role of *H. pylori* infection in chronic cholecystitis. During the formation of gallstones, there are many etiological factors. Also, among them, infection, inflammation, and the immune system play a vital role [12]. Recently, increasing studies pointed that the gut microbiome participated in gallstone formation by reducing bile acid metabolism and affecting the dynamic equilibrium [13, 14]. *H. pylori* is one of them.

Therefore, a series of studies have been carried out to find the relationship between gallbladder *H. pylori* infection and chronic cholecystitis/cholelithiasis [15–34]. Karagin et al. [28] supported the role of *H. pylori* in the development of cholecystitis in humans. Also, Dar et al. [32] pointed an association between the presence of *H. pylori* and hepatobiliary stone diseases. However, in a study conducted by Fallone et al. [35], they found no *H. pylori* present in any of the patients diagnosed with gallstones, thus raising doubt as to the possible association between *H. pylori* and gallstone diseases.

Consequently, the relationship between *H. pylori* infection in the gallbladder and cholecystitis/cholelithiasis still remains unclear, with controversial results reported in various studies. We perform this meta-analysis aiming to review data from related studies to evaluate the potential association between *H. pylori* infection in the gallbladder and chronic cholecystitis/cholelithiasis.

## 2. Methods

This systematic review and meta-analysis were conducted based on the Meta-Analysis of Observational Studies in Epidemiology (MOOSE) statement guidelines [36].

### 2.1. Search Strategy

We searched for relevant studies from PubMed, Embase, and Cochrane databases. The last search was in July 2020. The retrieval strategy used was as follows: ((*Helicobacter pylori*) or (*Campylobacter pylori*) OR (*H. pylori*) OR (*HP*) OR (*Helicobacter*) OR (*Helicobacter bilis*) OR (*Helicobacter hepaticus*) OR (*Helicobacter pullorum*) OR (*H. bilis*) OR (*H. hepaticus*) OR (*H. pullorum*) OR (*H. ganmani*) OR (*Helicobacter* species) OR (*Helicobacter* sp.) OR (*Helicobacter genus*) OR *Campylobacter* OR (*Campylobacter* infection) OR *Campylobacteriosis* OR (*Helicobacter pylori* infection) OR (*Helicobacter* infection) OR (*pylori*) OR (*enterohepatic Helicobacter* spp.) OR (*Campylobacter* spp.)) AND (cholelithiasis or cholecystolithiasis or gallstone^*∗*^ OR gall^*∗*^stone^*∗*^ OR (gallbladder AND stone^*∗*^) OR (gallbladder AND cholelith) OR (gallbladder AND lithiasis) OR bilestone^*∗*^ OR (bile AND stone^*∗*^) OR (bile AND lithiasis) OR (bile AND cholelith) OR (biliary AND calculus) OR (biliary AND stone^*∗*^) OR (biliary AND cholelith) OR (biliary AND lithiasis) OR cholecystitis). We also supplemented eligible studies from the references in the review articles by manual retrieval.

### 2.2. Eligibility Criteria

The inclusion criteria are as follows: (1) original cross-sectional studies, cohort studies, or case-control studies which are about the relationship between chronic cholecystitis/cholelithiasis and *Helicobacter* species infection of the gallbladder, (2) papers in which the method of detecting *Helicobacter* species infection was polymerase chain reaction (PCR), (3) papers that provide sufficient information to calculate odds ratios (ORs) and 95% confidence intervals (CIs), and (4) published in English as a full paper before July 2020.

The exclusion criteria are as follows: (1) reviews, meta-analysis, letters, commentaries, case reports, and animal studies, (2) studies that had no control group or inappropriate control group, and (3) duplicate study samples.

### 2.3. Data Extraction and Quality Assessment

Two investigators extracted the following data from each study in a standardized manner, independently: first author's name, publication year, country, study population and demographic data (e.g., gender and age), biliary diseases, the detection method for *Helicobacter* species, sample, specific primer, *Helicobacter* species, as well as the number of cases and controls.

The quality of each study included was also independently assessed by two investigators according to the 9-star Newcastle–Ottawa quality assessment scale [37], the content of which is as follows: (1) the selection of cases and controls, (2) the comparability of cases and controls, and (3) the assessment of exposure.

Any discrepancies on data extraction or quality assessment were resolved through discussion.

### 2.4. Statistical Analysis

All statistical analyses were conducted with Stata statistical software (version 12.0; College Station, Texas 77845, USA) by two authors, independently. Also, we used a random-effect model to calculate the summarized ORs with 95% CIs considering the diversity among various studies due to different study designs, methodologies, and populations.

The standard chi-square test was performed to assess statistical heterogeneity between studies, and the result was quantified by *I*^2^. Values of *I*^2^ < 25%, 25–50%, and >75% indicate low, medium, and high heterogeneity, respectively. When highly heterogeneous outcome was observed, sensitivity analysis was performed to investigate the influence of the individual study and the stability of results.

Because of the regional differences in the prevalence of *H. pylori* infection [38], we also conducted a subgroup analysis of studies from Asian and non-Asian countries in order to reduce regional interference in the study. We also performed subgroup analyses of samples and primers for detecting *H. pylori*.

### 2.5. Assessment of Bias and Sensitivity Analysis

Publication bias was assessed by funnel scatter-plots, Begg's adjusted rank correlation, and Egger's regression asymmetry tests. Publication bias will lead to asymmetry in funnel plots. Also, for Begg's and Egger's test, *P* < 0.05 was considered to indicate potential publication bias.

As for sensitivity analysis, leave-one-out sensitivity and stratified analysis were also carried out to investigate the influence of the individual study and the stability of results.

## 3. Results

### 3.1. Description of the Studies

**Figure 1:**
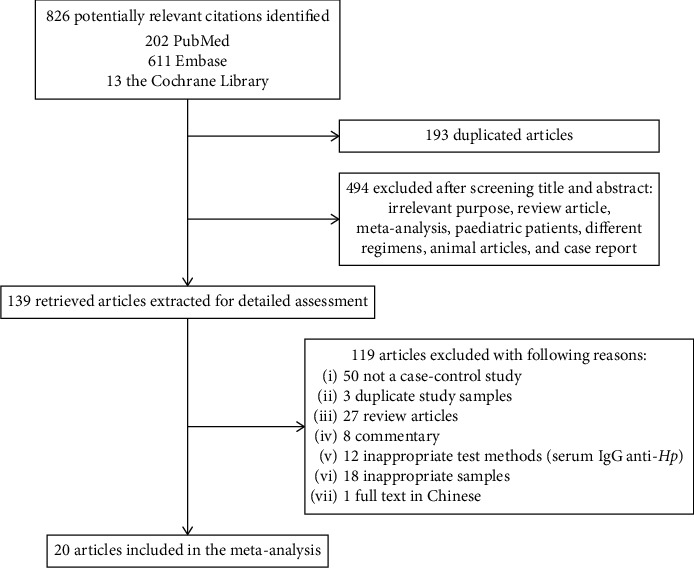
Flow diagram of the systematic review of the literature.

The search strategy yielded 826 publications, of which 202 were from PubMed, 611 were from Embase, and 13 were from the Cochrane Library. Of these records, 193 were excluded as duplicate articles and 494 were excluded as irrelevant on the basis of title and abstract. 139 were reviewed for detailed assessment. Of these publications, 119 were excluded from our final analysis, and the reasons are listed in Figure 1. Besides, 2 articles by Bulajic et al. [16, 17] were included in our meta-analysis due to the independence of the sample. Eventually, 20 studies, published between 2000 and 2018, were included in our meta-analysis.

The main characteristics of the included studies are listed in Table 1. The selected 20 studies included a total size of 1735 participants, of which 1197 were for the case group and 538 were for the control one. Of these studies, 11 of them were conducted in Asia (Japan, India, Thailand, Iran, Pakistan, and Russia) and 9 studies were conducted in non-Asian areas (German, Serbia, Brazil, Greece, Sweden, New Zealand, and Netherlands). There were totally 5 species of *Helicobacter* species including *H. pylori*, *H. bilis*, *H. hepaticus*, *H. pullorum*, *and H. ganmani* in our study. *H. pylori* was the commonest species which was identified in 17 studies. *H. bilis* was reported in 2 studies. *H. hepaticus*, *H. pullorum*, *and H. ganmani* each were reported in 1 study. Among the included studies, 12 studies used bile as sample, 5 studies used the gallbladder as sample, 2 studies used bile and the gallbladder as sample, and only 1 study used gallstone as sample. The specimens of bile, gallbladder tissue, and gallstone were obtained during cholecystectomy, endoscopic retrograde cholangiopancreatography (ERCP), percutaneous transhepatic cholangio-drainage, endoscopic papillotomy, and regular operation of gastrectomy or hepatectomy. Polymerase chain reaction (PCR) was used to detect *Helicobacter* species infection, and the positive rate is 344/1735. The primer of target genes in the included studies varied. The 16S rRNA gene was the most commonly used primer and had been used in 12 studies. Urease gene was used as primer in 3 studies, and 26 kDa species-specific protein gene was used in 1 study. 3 other studies used two primers in combination.

### 3.2. Study Quality

Each study chosen for this meta-analysis was carefully assessed according to the Newcastle–Ottawa scale assessment. All studies obtained high scores, defined as ≥6 stars (Table 1).

### 3.3. The General Analysis

**Figure 2:**
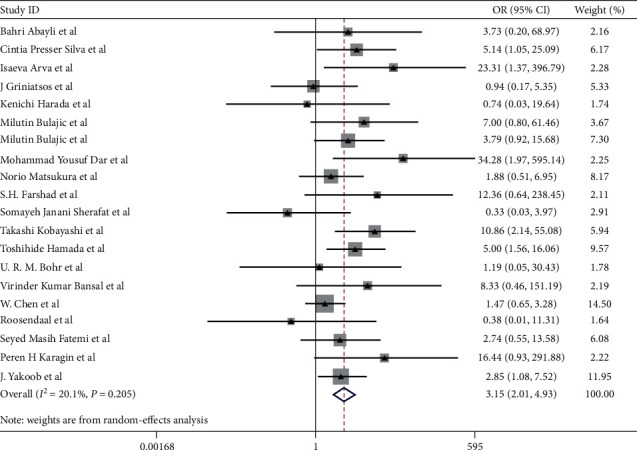
Pooled ORs for the relationship between *Helicobacter* species infection of the gallbladder and chronic cholecystitis and cholelithiasis. The areas of the squares are proportional to the study sample sizes.

Overall, as shown in Figure 2, the pooled OR for patients with chronic cholecystitis/cholelithiasis compared with participants without chronic cholecystitis/cholelithiasis was 3.15 (95% CI, 2.01–4.93), indicating a potential positive association between gallbladder *Helicobacter* species infection and chronic cholecystitis/cholelithiasis risk. Also, there was low heterogeneity (*I*^2^ = 20.1%, *P*=0.205).

Besides, we found a higher gallbladder *H. pylori* infection rate in the chronic cholecystitis/cholelithiasis group than that in the control group in 17 studies that focused on *H. pylori* (OR = 3.05; 95% CI, 1.81–5.14) (Table 2). For other *Helicobacter* species, studies concerned were limited (Table 2). In addition to *H. pylori*, a higher *H. hepaticus* infection rate in the chronic cholecystitis/cholelithiasis than that in the controls was also observed (41.7% vs. 12.5%, *P*=0.007). Although the prevalence of *H. bilis* was also higher in chronic cholecystitis/cholelithiasis (21.3% vs. 10.3%, *P*=0.273), the results had no statistically significant difference. *H. pullorum and H. ganmani* infection rates were low in both cases and controls (6.0% vs. 0.0%, *P*=0.073; 1.8% vs. 0.0%, *P*=0.914, respectively).

### 3.4. Subgroup Analysis for *H. pylori* Infection

**Figure 3:**
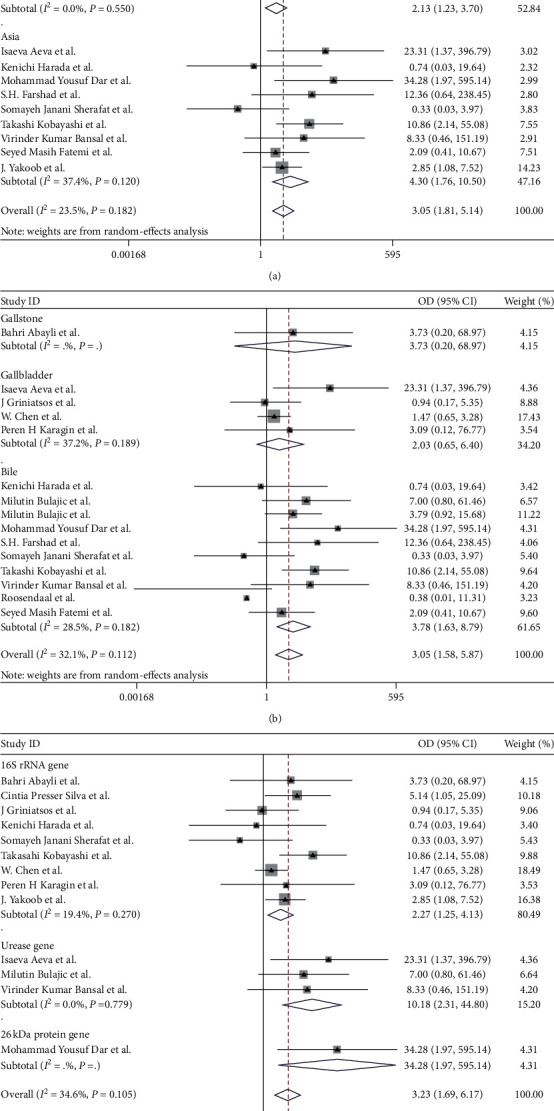
Pooled ORs for the relationship between *H. pylori* infection of the gallbladder and chronic cholecystitis and cholelithiasis: (a) subgroup based on country; (b) subgroup based on sample; (c) subgroup based on specific primer. The areas of the squares are proportional to the study sample sizes.

For *H. pylori* infection, subgroup analyses were conducted according to country, sample, and specific primer in Figure 3. Compared with overall results, there were no significant differences in subgroup studies. Gallbladder *H. pylori* infection was also positively correlated with the risk of chronic cholecystitis/cholelithiasis in both Asian and non-Asian groups. The ORs for Asian and non-Asian country studies were 4.30 (95% CI, 1.76–10.50; *I*^2^ = 37.4%) and 2.13 (95% CI, 1.23–3.70; *I*^2^ = 0.0%), respectively. In bile samples, the pooled OR was 3.78 (95% CI, 1.63–8.79), indicating a significantly higher detection rate in the chronic cholecystitis/cholelithiasis patients than that in the control group. In gallbladder samples, although the pooled OR was 2.03 (95% CI, 0.65–6.40), the result had no statistically significant difference (*P*=0.226). Only 1 study used the gallstone as the sample, and the result had no statistically significant difference (*P*=0.377). The ORs of 16S rRNA gene and urease gene were 2.27 (95% CI, 1.25–4.13) and 10.18 (95% CI, 2.31–44.80), respectively. Only 1 study used 26 kDa protein gene.

### 3.5. Assessment of Bias and Sensitivity Analysis

**Figure 4:**
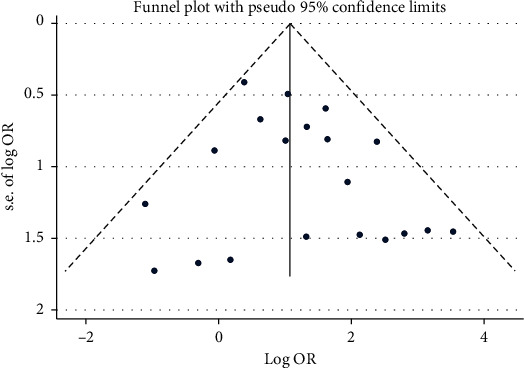
Funnel plot. The distribution of dots is relatively symmetrical, suggesting no strong evidence or publication bias.

As shown in Figure 4, funnel plot is relatively symmetrical, suggesting no strong evidence for publication bias. In addition, there was no evidence for bias using either Begg's method (*P*=0.974) or Egger's weighted regression method (*P*=0.268).

All the results remained significant when leave-one-out sensitivity analyses were conducted, indicating the stability of results for this meta-analysis.

## 4. Discussion

Since *Helicobacter* species was detected from the gallbladder tissue, gallstone, and bile taken from the gallbladder, numerous studies have shown that *Helicobacter* species, especially *H. pylori*, may contribute to the formation of cholesterol gallstones [39]. However, research studies conducted so far have failed to establish a definite relationship between *H. pylori* infection and gallstone diseases. Our systematic review and meta-analysis is to assess the relationship between *H. pylori* infection in the gallbladder and chronic cholecystitis/cholelithiasis.

Overall, our research showed that the prevalence of *Helicobacter* species in the chronic cholecystitis/cholelithiasis group was significantly higher than that in the control group (24.40% vs. 8.55%, *P*=0.000), suggesting a potential positive correlation between *Helicobacter* species infection in the gallbladder and increased risk of chronic cholecystitis/cholelithiasis. Likewise, a higher gallbladder *H. pylori* infection rate was found in the chronic cholecystitis/cholelithiasis group than that in the control group (23.70% vs. 7.23%, *P*=0.000). Due to the small size of relevant studies, there was no significantly positive correlation between other *Helicobacter* species infection in the gallbladder with gallstone diseases except for *H. hepaticus*.

People in countries with lower socioeconomic status are more likely to be infected with *H. pylori*. Previous studies [38] reported that the prevalence of *H. pylori* in Asia, Africa, and South America is higher than that in North America, Western Europe, and Australia, which may influence *H. pylori* infection rate in the gallbladder. So we divided the subgroups according to whether they were Asian countries or not. This positive association still persisted both in Asian or non-Asian countries. Also, the OR of Asian countries is quite higher than that of non-Asian countries (4.30 and 2.13, respectively). Different samples and different specific primers of PCR in each study included may also affect the results, so we performed subgroup based on the sample and specific primer. The *H. pylori* infection rate in bile was higher in chronic cholecystitis/cholelithiasis than that in controls (OR = 3.78, *P*=0.002), indicating that *H. pylori* is relatively more colonized in bile in gallstone diseases. Unfortunately, there was no significant difference in the prevalence of *H. pylori* infection in the gallbladder. The OR of 16S rRNA was lower than that of urease gene (2.27 and 10.18, respectively). Maybe false-positive result caused by urease A or C gene cross-reacting with the urease gene of other organisms accounted for it [40].

In addition to ethnicity and genetics, advancing age and female gender as well as obesity are also risk factors for gallstones [5]. The risk of gallstone diseases increases with age in all racial groups. Because of female sex hormones and increased cholesterol secretion, females with obesity are more likely to get gallstones [41]. However, Bohr et al. [25] found that the most significant risk factors for gallstone disease were patients with age >65 years and being overweight. Female gender was not statistically significant. Moreover, the infection rate of *H. pylori* was also associated with age [38]. Bulajic et al. [16] noted that the possibility of detecting *H. pylori* in the bile increased steadily with increasing age. High *H. pylori* infection may affect the prevalence of gallstones diseases. *H. pylori* infection is less related to gender. In a marching cohort conducted by Miendje Deyi et al. [42], the proportion of *H. pylori* infected patients was virtually the same in males and females. However, due to limited studies and insufficient data of age, gender, and body mass index (BMI), we did not evaluate their impact on chronic cholecystitis/cholelithiasis. Further studies were suggested to clarify the role of age, gender, and BMI played in gallstones diseases.

The pathways of *H. pylori* into bile have not been completely explained, one possibility is the translocation from the duodenum via Oddi's sphincter, and another is penetration via the portal circulation and lymphatic vessels [43, 44]. Bulajic et al. [16] and Bansal et al. [30] reported that there was a strong correlation between the presence of *H. pylori* in the bile and in the stomach, which may testify to the above views. In a study in Thailand [45], nevertheless, they suggested that gastroduodenal and hepatobiliary infections were caused by different *H. pylori* strains based on PCR analysis.

The relationship between *H. pylori* infection and gallstones may have several potential mechanisms. Firstly, *H. pylori* can produce oxidative stress and free radical reactions through the reactive oxygen species (ROS) and reactive nitrogen species (RNS) system [46, 47] and release large amounts of proinflammatory and vasoactive substances, such as interleukins (IL)-1, IL-6, and tumor necrosis factor (TNF)-*α* [48], which are involved in gallbladder inflammatory disorders and pathogenesis of gallstones. Secondly, *H. pylori* may also promote the risk of stone formation by acting as a foreign body nidus [49].Thirdly, urease-positive *Helicobacter* species are capable of precipitating calcium and participating in initiating gallstone formation [50]. Fourthly, *H. pylori* induces beta-glucuronidase, bacterial hydrolase, and phospholipase to deconjugate bile and hydrolyze phosphatidylcholine, thus facilitating the synthesis of free bilirubin, free bile acids, and free fatty acids. Free ionized calcium ion precipitates with free bilirubin to form calcium bilirubinate. Calcium bilirubinate, free bile acids, and free fatty acids are the major components of brown pigment stones [13].

Since *H. pylori* infection promotes the formation of gallstones, we consider whether *H. pylori* eradication therapy can prevent the formation of gallstones. In the studies of Zhang et al. [51] and Takahashi et al. [52], they both found a statistically significant trend in the reduction of gallstones among the patients who received *H. pylori* eradication. However, the drawback is that they used the fasting ^13^C urea breath test and serum anti-*Hp* antibody test as detection methods for *H. pylori*. Although the two methods are highly specific and sensitive, they cannot locate *H. pylori* infection in the gallbladder, which may undermine the reliability of the study. Besides, the serological test is not a reliable test for evaluating eradication therapy because antibodies can be present in the blood for a long time even after successful eradication [53]. Also, the presence of gallstones in the gallbladder was diagnosed by abdominal ultrasonography, which may lead to a false-negative result. In a word, the exact effect of eradicating *H. pylori* infection to prevent gallstone formation remains to be elucidated.

It is noteworthy that our meta-analysis also has some strengths. Firstly, in order to avoid the influence of different test methods on the research, we selected studies using PCR as the *H. pylori* infection detection method, which is a highly sensitive and specific method. Serological testing is excluded for the relatively high negative predictive value [54–56]. Besides, there was no gallbladder disease in the control group. Due to the high correlation between *H. pylori* infection and gallbladder cancer, these patients are excluded [57, 58]. There was a previous meta-analysis which assessed the association between *H. pylori* infection and chronic cholecystitis/cholelithiasis [59]. However, the included studies mixed *H. pylori* and other *Helicobacter* species. In our study, we analyzed *H. pylori* and other *Helicobacter* species separately and conducted more in-depth subgroup analyses of *H. pylori* based on the extracted sample and specific primer.

However, several limitations of this meta-analysis should be noted. Most patients with cholelithiasis have been treated with antibiotics. Different doses and courses of antibiotics might inhibit the growth of the bacterium and influence the results. Besides, since the retrograde contamination is caused by ERCP, there may be false-positive results. Furthermore, there are diverse bacterial communities in human bile samples such as *Pseudomonas aeruginosa*, *E. coli*, *Klebsiella pneumoniae*, *Enterococcus* spp., and *Acinetobacter* spp. Tajeddin et al. [60] pointed that *Escherichia coli* was significantly related to gallstone formation. Our study cannot avoid interference from other bacteria. Previous studies have suggested that *H. pylori* infection may be associated with pancreatic cancer [61], liver cirrhosis, and liver cancer [62]. It is a pity that the control group could not completely exclude such patients in our study.

In conclusion, our meta-analysis showed a significant positive correlation between *H. pylori* infection in the gallbladder and increased risk of cholelithiasis and chronic cholecystitis, especially in Asian countries. The association was more pronounced using the bile sample and urease gene primer. Also, more studies are needed to determine whether age, gender, and BMI interfere with the relationship between gallbladder *H. pylori* infection and chronic cholecystitis/cholelithiasis. It is hoped that more randomized trials in the future can better clarify the relationship between them and provide clinical evidence for the prevention and treatment of chronic cholecystitis/cholelithiasis.

## Figures and Tables

**Table 1:** Main characteristics of the included studies in this meta-analysis.

Author	Year	Country	Gender, M/F	Age, years (mean age ± SD) (range age)	Biliary diseases	Samples	Specific primers	*Helicobacter* species	Cases	Controls	Nos.
Bahri Abayli et al.	2005	Turkey	28/49	M: 50 (25–73) F: 54 (24–80)	Cholelithiasis, cholecystitis	Gallstone	16S rRNA gene	*H. pylori*	77	20	6
Cíntia Presser Silva et al.	2003	Brazil	27/37	Cases: 51.5 ± 16.4 (21–80); controls: 38.3 ± 17.4 (18–70)	Cholelithiasis	Bile, gallbladder	16S rRNA gene	*H. pylori*	46	18	7
Isaeva Aeva et al.	2018	Russia	37/77	Cases: cholecystitis 42.6 ± 4.8, cholelithiasis 57.1 ± 3.6; controls: 42.9 ± 3.4	Cholelithiasis, cholecystitis	Bile ducts, gallbladder	Urease C gene	*H. pylori*	84	30	6
Griniatsos et al.	2009	Greece	22/67	63 (51–73)	Cholelithiasis	Gallbladder	16S rRNA gene	*H. pylori*	89	42	7
Kenichi Harada et al.	2001	Japan	None	None	Cholelithiasis	Bile	16S rRNA gene	*H. pylori*	39	9	6
Milutin Bulajic et al.	2002	Serbia	32/40	56.7 ± 16.45	Cholelithiasis	Bile	Urease A gene	*H. pylori*	65	7	6
Milutin Bulajic et al.	2002	Serbia	42/47	None	Cholelithiasis	Bile	Urease A gene, 16S rDNA gene	*H. pylori*	63	11	7
Mohammad Yousuf Dar et al.	2016	India	29/46	Cases: 40.5 ± 10.85 (18–75); controls: 37.3 ± 9.6 (18–54)	Cholelithiasis	Bile	26 kDa surface antigen	*H. pylori*	50	25	6
Norio Matsukura et al.	2002	Japan, Thailand	30/15	65.2 (24–83)	Cholelithiasis, cholecystitis	Bile	16S rRNA gene	*H. bilis*	42	14	7
Farshad et al.	2004	Iran	None	Cases: 18–70; controls: 20–67	Cholelithiasis, cholecystitis	Bile	16S rRNA gene, isocitrate dehydrogenase gene	*H. pylori*	33	40	6
Somayeh Janani Sherafat et al.	2012	Iran	None	None	Cholelithiasis	Bile	16S rRNA gene	*H. pylori*	74	13	6
Takashi Kobayashi et al.	2005	Japan	None	None	Cholelithiasis	Bile	16S rRNA gene	*H. pylori*	30	21	7
Toshihide Hamada et al.	2009	Japan	43/49	Cases: 61.2 controls: 69.2	Cholelithiasis, cholecystitis	Bile	16S rRNA gene	*H. hepaticus*	60	32	6
Bohr et al.	2007	German	None	None	Cholelithiasis	Gallbladder	16S rRNA gene	*H. ganmani*	57	22	7
Virinder Kumar Bansal et al.	2012	India	None	None	Cholelithiasis, cholecystitis	Bile	Urease A gene	*H. pylori*	49	12	7
Chen et al.	2003	New Zealand	39/68	Cases: symptomatic gallstones 47.4 (23.3–79.9); asymptomatic gallstones 50.6 (35.7–66.0); controls: 58.7 (23.2–74.4)	Cholelithiasis	Gallbladder	16S rRNA gene	*H. pylori*	70	37	6
Roosendaal et al.	2002	Netherlands	None	73 (42–85)	Cholelithiasis	Bile	16S rDNA gene	*H. pylori*	28	3	7
Seyed Masih Fatemi et al.	2017	Iran	58/19	46.85 ± 14.53	Cholelithiasis, cholecystitis	Bile	Hsp60 gene, 16S rRNA gene	*H. pylori*, *H. bilis*	52	25	7
Karagin et al.	2010	Sweden	79/113	Cases: 48 (20–84); controls: 58 (11–85)	Cholecystitis	Gallbladder	16S rRNA gene	*H. pylori*, *H. pullorum*	100	102	6
Yakoob et al.	2011	Pakistan	43/101	49 ± 14 (18–84)	Cholecystitis, cholelithiasis	Bile, gallbladder	16S rRNA gene	*H. pylori*	89	55	6

M, male; F, female; NOS, Newcastle–Ottawa scale assessment.

**Table 2:** Prevalence of *Helicobacter* species in this meta-analysis.

*Helicobacter* species	Positive in cases	Positive in controls	OR (95% CI)	*P* value
*H. pylori*	246/1038	34/470	3.05 (1.81–5.14)	0.000
*H. pullorum*	6/100	0/102	14.10 (0.78–253.71)	0.073
*H. ganmani*	1/57	0/22	1.19 (0.05–30.43)	0.914
*H. hepaticus*	25/60	4/32	5.00 (1.56–16.06)	0.007
*H. bilis*	20/94	4/39	1.96 (0.59–6.55)	0.273

OR, odds ratio; CI, confidence interval.

## Data Availability

The data supporting the current study are available from the corresponding author upon request.

## Abbreviations

*H. pylori*: *Helicobacter pylori*
*Hp*: *Helicobacter pylori*
MALT: Gastric malignant lymphoma of the mucosa-associated lymphoid tissue
PCR: Polymerase chain reaction
ERCP: Endoscopic retrograde cholangiopancreatography
BMI: Body mass index
ROS: Reactive oxygen species
RNS: Reactive nitrogen species
IL: Interleukins
TNF: Tumor necrosis factor.
