# Corrigendum to “The Relationship between *Helicobacter pylori* Infection of the Gallbladder and Chronic Cholecystitis and Cholelithiasis: A Systematic Review and Meta‐Analysis”

**DOI:** 10.1155/cjgh/9835917

**Published:** 2025-08-29

L. Wang, J. Chen, W. Jiang, et al., “The Relationship between *Helicobacter pylori* Infection of the Gallbladder and Chronic Cholecystitis and Cholelithiasis: A Systematic Review and Meta‐Analysis,” *Canadian Journal of Gastroenterology and Hepatology* 2021 (2021): 8886085, https://doi.org/10.1155/2021/8886085.

In the article, there were errors in the third listed affiliation. The corrected affiliation list is shown below and has been corrected in the article.

Liang Wang,^1,2^ Junyin Chen,^1,3^ Wenxi Jiang,^1^ Li Cen,^1^ Jiaqi Pan,^1^ Chaohui Yu,^1^ Youming Li,^1^ Weixing Chen,^1^ Chunxiao Chen,^1^ and Zhe Shen^1^

^1^Department of Gastroenterology, The First Affiliated Hospital, College of Medicine, Zhejiang University, Hangzhou, China

^2^Endoscopy Center, Cangzhou Central Hospital of Hebei Province, Cangzhou, Hebei Province, China

^3^Department of Gastroenterology, Affiliated Hospital of Shaoxing University, Shaoxing, Zhejiang Province, China

We apologize for this error.

